# The role of central amygdaloid nucleus in regulating the nongenomic effect of aldosterone on sodium intake in the nucleus tractus solitary

**DOI:** 10.1002/brb3.2615

**Published:** 2022-05-19

**Authors:** Rui Wang, Nan Wang, Wenhui Liang, Tingting Lin, Hu Qiao

**Affiliations:** ^1^ Key Laboratory of Shaanxi Province for Craniofacial Precision Medicine Research College of Stomatology Xi'an Jiaotong University Xi'an China; ^2^ Department of Human Anatomy Shaanxi University of Chinese Medicine Xi'an China

**Keywords:** aldosterone, CeA, nongenomic effect, sodium intake

## Abstract

**Objective:**

The central nucleus of the amygdala (CeA) has dense downward fiber projections towards the nucleus tractus solitary (NTS) and can modulate the activity of NTS taste neurons. However, whether CeA affects the nongenomic role of aldosterone (ALD) in regulating sodium intake at the NTS level remains unclear.

**Methods:**

First, 40 adult male Sprague Dawley rats were divided into five groups, referring to different concentrations of ALD, to observe the sodium intake pattern compared with the vehicle (*n* = 8). ALD, the mineralocorticoid receptor antagonist spironolactone (SPI), and ALD + SPI were injected into the NTS. Then, the rats were divided into four groups (*n* = 16): bilateral/unilateral CeA electrolytic lesions, bilateral/unilateral CeA sham lesions. After recovery, one stainless steel 23‐gauge cannula with two tubes was implanted into the rat NTS, and all rats underwent a recovery period of 7 days. Then, each group was divided into two subgroups that received aldosterone or control solution injection, and the cumulative intake of 0.3 mol/L NaCl solution was recorded within 30 min.

**Results:**

Bilateral CeA lesion eliminated the increased 0.3 mol/L NaCl intake induced by aldosterone microinjected into the NTS (CeA lesion: 0.3 ± 0.04 ml/30 min vs. sham lesion: 1.3 ± 0.3 ml/30 min). Unilateral CeA lesion reduced the increased NaCl intake induced by aldosterone microinjected into the NTS compared with the control group (*p* < .05) in the first 15 min but not in 15–30 min (*p* > .05). In sham lesion rats, aldosterone (5 ng/0.1 μl) still induced a significant increase in NaCl intake (aldosterone: 1.3 ± 0.3 ml/30 min vs. control: 0.25 ± 0.02 ml/30 min) (*p* < .05).

**Conclusion:**

The results verified that the complete CeA may play an important role in aldosterone to regulate the nongenomic effect on rapid sodium intake.

## Graphical abstract

1. ALD can regulate sodium uptake through non‐genomic effects.
2. Unilateral CeA lesions partially offset the sodium intake increase effect of ALD.
3. Bilateral CeA lesions significantly offset the sodium intake increase effect of ALD.

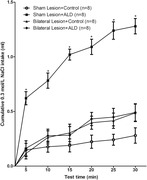

## 1 INTRODUCTION

Sodium appetite is a behavioral response initiated by a salty taste and is the result of multiple types of afferent information modulated and integrated through different levels of neuroendocrine systems. The renin–angiotensin–aldosterone system plays an essential role in the regulation of sodium appetite in which aldosterone regulates sodium excretion in the kidney. It is also involved in the management of salty perception and sodium intake through the peripheral mechanism of regulation of sodium channels in the tongue epithelium and the influence of sodium‐sensitive neurons in the central nervous system. Formenti et al. reported that sodium intake was significantly increased in a dose‐dependent manner following long‐term and chronic treatment using aldosterone in the fourth ventricle of rats (Formenti et al., 2013), observations supported by the research of Koneru et al., who found that this phenomenon could be inhibited by shRNA interference of mineralocorticoid receptors in the solitary nucleus (Koneru et al., 2014). Thus, aldosterone has been demonstrated to have far‐reaching effects on the intake of sodium in the solitary nucleus (NTS). In addition to classical genomic effects, aldosterone may also operate diffusely and rapidly in a nongenomic manner in vivo (Williams, 2013). Aldosterone can bind to mineralocorticoid receptors (MRs) in the cytoplasm to form an MR–aldosterone complex, and then the complex is translocated to the nucleus, thereby exerting the regulation of renal water reabsorption through genomic effects. Notably, the first effect usually occurs 30–60 min after aldosterone release/intake. Aldosterone can also function through nongenomic effects. Nongenomic effects can increase second messengers of membrane signaling transduction pathways and thereby may exert a rapid effect at the membrane level (within minutes) without transcription or protein synthesis. Recently, our laboratory reported that injection of different doses of aldosterone into the solitary nucleus induced fast sodium intake in rats (Qiao et al., 2016).

In another respect, the central amygdaloid nucleus (CeA) represents a vital subnucleus in the amygdaloid nucleus and participates in the regulation of multiple physiological functions. For example, the CeA has complex neural links via projections with other regions of the brain (Fadok et al., 2018; Smith et al., 2016; Wang et al., 2018). The CeA receives ascending fiber projections of numerous nuclei in the brainstem, such as the solitary nucleus, paribral nucleus, dorsal nucleus of the vagus nerve, and locus coeruleus. The CeA also emits fiber projections to the solitary nucleus, paribral nucleus, dorsal nucleus of the vagus nerve, and periaqueductal gray matter. Recent studies have demonstrated that the CeA is an important structure for the integration of information in the peripheral forebrain in addition to being essential for the regulation of the transmission of information about salt in the brainstem, which has close fibrous connections with a number of related brain regions that regulate the behavior of sodium intake. Numerous injury experiments and neuropharmacological studies have demonstrated that CeA affects sodium intake and the reabsorption and excretion of sodium in the kidney, thereby regulating sodium balance in the body.

Thus, the CeA is an important neural structure that regulates sodium intake and affects neuroregulation at the medullary level. However, it remains unclear whether CeA influences the regulation of the nongenomic effects of sodium intake by aldosterone in the solitary nucleus. Hence, based on observations from previous research, the influence of CeA on aldosterone's nongenomic effects on sodium intake in the solitary nucleus of rats was investigated in the present study, in addition to descending modulation mechanisms.

## 2 MATERIALS AND METHODS

### 2.1 Animals

Adult male Sprague Dawley rats with a body weight of 250 ±20 g were used in the present study and were provided by the Medical Experimental Animal Centre of Xi'an Jiaotong University (Xi'an, China). The NIH Guide for the Care and Use of Laboratory Animals was observed when planning animal studies, which received approval from the Institutional Animal Care Committee of Xi'an Jiaotong University. The rats were single‐housed unless otherwise stated, first prior to experimentation for adaptation for 1 week, and then were submitted to practice infusion and maintained at a humidity of 55 ± 10% and temperature of 23 ± 2℃, within a 12/12 h light/dark cycle from 08:00 to 20:00. The rats had free access to food and drinking water. All experimental protocols for rats used in the present study were performed in accordance with guidelines established by the Institutional Animal Care and Use Committee of Xi'an Jiaotong University. Through our best efforts, the number of experimental animals was minimized, and pain during experimentation was alleviated where possible.

### 2.2 Materials and instruments

Aldosterone was purchased from Sigma and dissolved in a solution of DMSO in H_2_O (at a volume ratio of 1:100). The same concentration of DMSO was used in the control group as a vehicle. A 0.1 μl volume of drugs was injected into the bilateral NTS. A final concentration of 5 ng/0.1 μl aldosterone was used for microinjection, as previously reported (Brailoiu et al., 2013; Formenti et al., 2013; Francis et al., 2001). The following instruments were used in this study: a 1‐μl microinjector (Hamilton), a stereotaxic apparatus (SN‐2N, Narishige), a concentric electrode (CEA 200, MicroProbes), a lesion‐making device (53500, Ugo Basile), metabolism/feeding‐drinking cages, part of a feeding‐drinking‐activity analyzer (41800111213, UGO), and a small animal anesthetic machine (RWD).

### 2.3 Electrolytic CeA lesions

Rats were anaesthetized with isoflurane (5% induction, 2% maintenance at 1 L/min) and then fixed in the stereotaxic apparatus, ensuring that the skull was level between the bregma and the lambda. After disinfection with alcohol, the fur and skin on the skull were removed, and the calvarium was exposed. The location of the CeA was ascertained based on a brain map of rats (2.5 mm caudal to bregma, 4.2 mm from the midline, and at a depth of 7.5 mm from the cortical surface). A microdrill was used to create a hole at the CeA to expose the dura mater. After removal of the fragments and dura mater, concentric electrodes were implanted bilaterally. Electrolytic lesions were created bilaterally at a constant current (400 μA for 25 s) at stereotaxic points above each side. A clip attached to the tail was used as an indifferent electrode. Sham lesion rats underwent the same surgical procedure and had an electrode placed at the same coordinates but without any current being passed. A lesion was created on a single side of the CeA by electrically stimulating only the left electrode, while the right electrode was disconnected from the output. All other steps of the surgery remained the same. Rats were allowed to recover for 3–5 days following creation of the lesion prior to the subsequent experiments being performed.

### 2.4 Implantation of cannulae in the solitary nucleus

Implantation of cannulae in the solitary nucleus was performed in rats 3–5 days after creation of electrolytic lesions in the CeA. Briefly, rats were anaesthetized and fixed in the stereotaxic apparatus, ensuring that the skull was level between the bregma and the lambda. After disinfection with alcohol, the fur and skin on the skull were removed, and the calvarium was exposed. The stereotaxic coordinates of the NTS were 13.9 mm caudal to the bregma, 0.5 mm lateral to the midline, and 7.8 mm below the surface of the skull. Each cannula was fixed with three screws and dental acrylic resin, and an obturator (30 gauge) was inserted. A prophylactic dose of penicillin was administered intramuscularly when the surgery was completed. All rats were allowed to recover for at least 7 days in metabolic cages following surgery, and a two‐bottle choice test was conducted synchronously during the recovery period. The metabolic cage is a component of the analyzer and measures food and water intake and monitors activity.

### 2.5 Microinjection

Drugs were microinjected into the NTS using a 1‐μl Hamilton syringe connected by PE‐10 polyethylene tubing to the 30‐gauge injection cannula (1 mm longer than the guide cannula). The injection volume into the NTS was 0.1 μl, with injection conducted over a 1‐min period followed by an additional 1 min with the injection cannula remaining in place. The injection cannula was then replaced with the obturator. Prior to the experiment officially beginning, rats were injected three times to ensure that they became accustomed to the injection process.

### 2.6 Behavioral tests

Rats were allowed to recover for 3 days following creation of electrolytic lesions and for 7 days after implantation of cannulae in the solitary nucleus prior to the start of the following experiments. Two‐bottle choice test training was conducted during the recovery.

1. Two‐bottle choice test: After the operation, the rats were placed in a metabolic cage for two‐bottle selection of distilled water and 0.3 mol/L NaCl for experimental training, freely eating and ingesting distilled water and 0.3 mol/L NaCl every day, once the intake of 0.3 mol/L NaCl was stabilized (usually 4–5 days), and then different drugs could be administered for experiments.
2. A dose of 5 ng/0.1 μl aldosterone or vehicle was injected into the bilateral NTS following creation of the electrolytic lesion in the bilateral CeA of rats, and its influence on the intake of 0.3 mol/L NaCl was recorded.

The rats were allocated into the electrolytic lesion or sham lesion group (*n* = 8 in each group), and half of the rats in each group were injected with 5 ng/0.1 μl aldosterone, while the other half were injected with vehicle. All rats were placed in metabolic cages after injection, and the cumulative intake of 0.3 mol/L NaCl solution within 30 min was automatically measured by the feeding‐drinking‐activity analyzer. Rats were fasted with food only within 30 min after administration.

3. A 5 ng/0.1 μl dose of aldosterone or vehicle was injected into the bilateral NTS after creation of the electrolytic lesion in the left CeA of rats, and its influence on the intake of 0.3 mol/L NaCl was analyzed.

The rats were divided into the electrolytic lesion and sham lesion groups (*n* = 8 in each group). Then, half of the rats in each group were injected with 5 ng/0.1 μl aldosterone, and the other half were injected with vehicle. The rats were placed in metabolic cages after injection, and the cumulative intake of 0.3 mol/L NaCl solution within 30 min was automatically measured with a feeding‐drinking‐activity analyzer. Rats fasted with food only within 30 min after administration.

### 2.7 Histological analysis

Immediately after the experiments, 2% Pontamine Sky Blue solution (0.2 μl) was injected into the NTS. The rats were then deeply anaesthetized with a high dose of chloral hydrate and perfused transcardially with PBS followed by 10% buffered formalin. In each rat, the brain was removed, fixed, frozen‐sectioned (40 μm thick) in the coronal plane, and analyzed under a light microscope to confirm the injection sites in the NTS, in accordance with the atlas of Paxinos and Watson. Coronal brain sections were cut at 40 μm thick and then Nissl stained to confirm the locations of lesions in the amygdala via microscopic inspection.

### 2.8 Data analysis

Statistical analysis was performed using a Statistical Program for Social Sciences statistical software (SPSS 18.0). All data are presented as means ± standard error of the mean (SEM) and were analyzed using two‐way analysis of variance with repeated‐measures. Post hoc comparisons were performed using Student–Newman–Keuls multiple comparison test. *p* < .05 was considered statistically significant. Taking into account Fitts's assumption, sodium intake was analyzed as cumulative and noncumulative data, and since there were no differences in the outcome of the hypothesis testing, such findings were presented as cumulative data as it is more usual.

## 3 RESULTS

### 3.1 Histology of localization analysis

**FIGURE 1:**
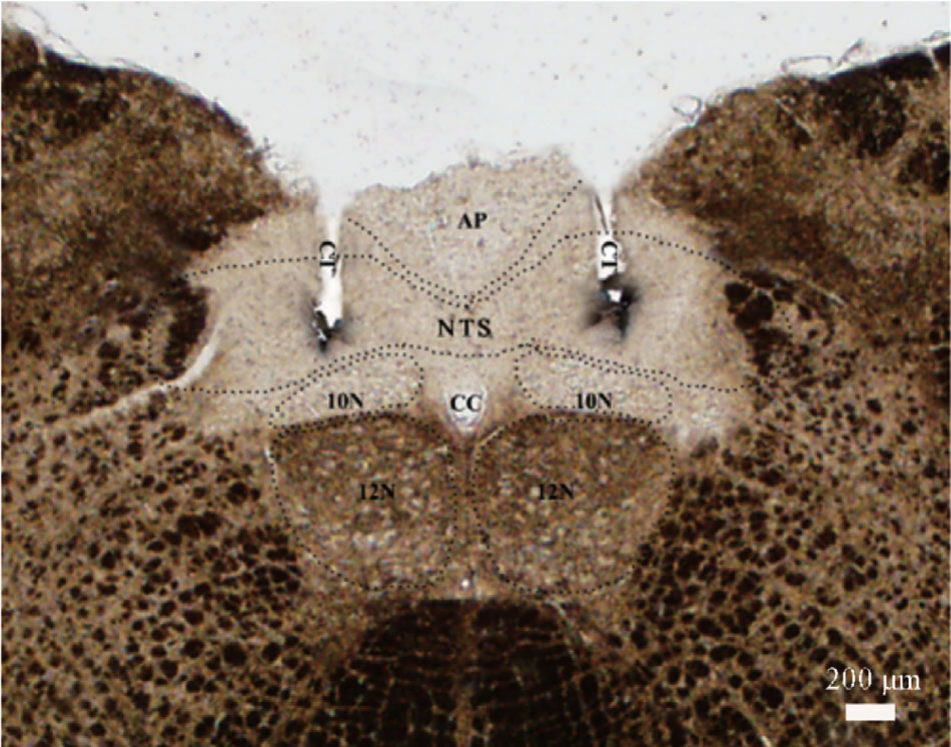
Photomicrograph illustrates bilaterally placed injections in the nucleus of the solitary tract (NTS). Injection sites on each side of the brain stem were identified by deposits of Pontamine Sky Blue dye. Scale bar = 200 μm. Abbreviations: AP, area postrema; CC, central canal; CT, cannula tract; 10N, dorsal motor nucleus of the vagus; 12N, hypoglossal nucleus

A total of 167 rats were used in this experiment; among those, 82 rats were used to verify the effect of ALD/SPI on sodium intake, and the other 70 rats were used to determine whether CeA can affect the nongenomic effect of ALD on sodium intake. Histologic localization indicated that among the 82 rats, the cannulae were placed accurately in 73 rats, and the majority of the injection sites were located in the middle of the solitary nucleus (Figure 1). The data gathered from these rats were used to analyze the effects of drug injection in the solitary nucleus regarding sodium intake. Data relating to rats that had histologically inaccurate localization were not significant compared with the vehicle treatment on sodium intake. Thus, the data were not included in the statistical analysis.

**FIGURE 2:**
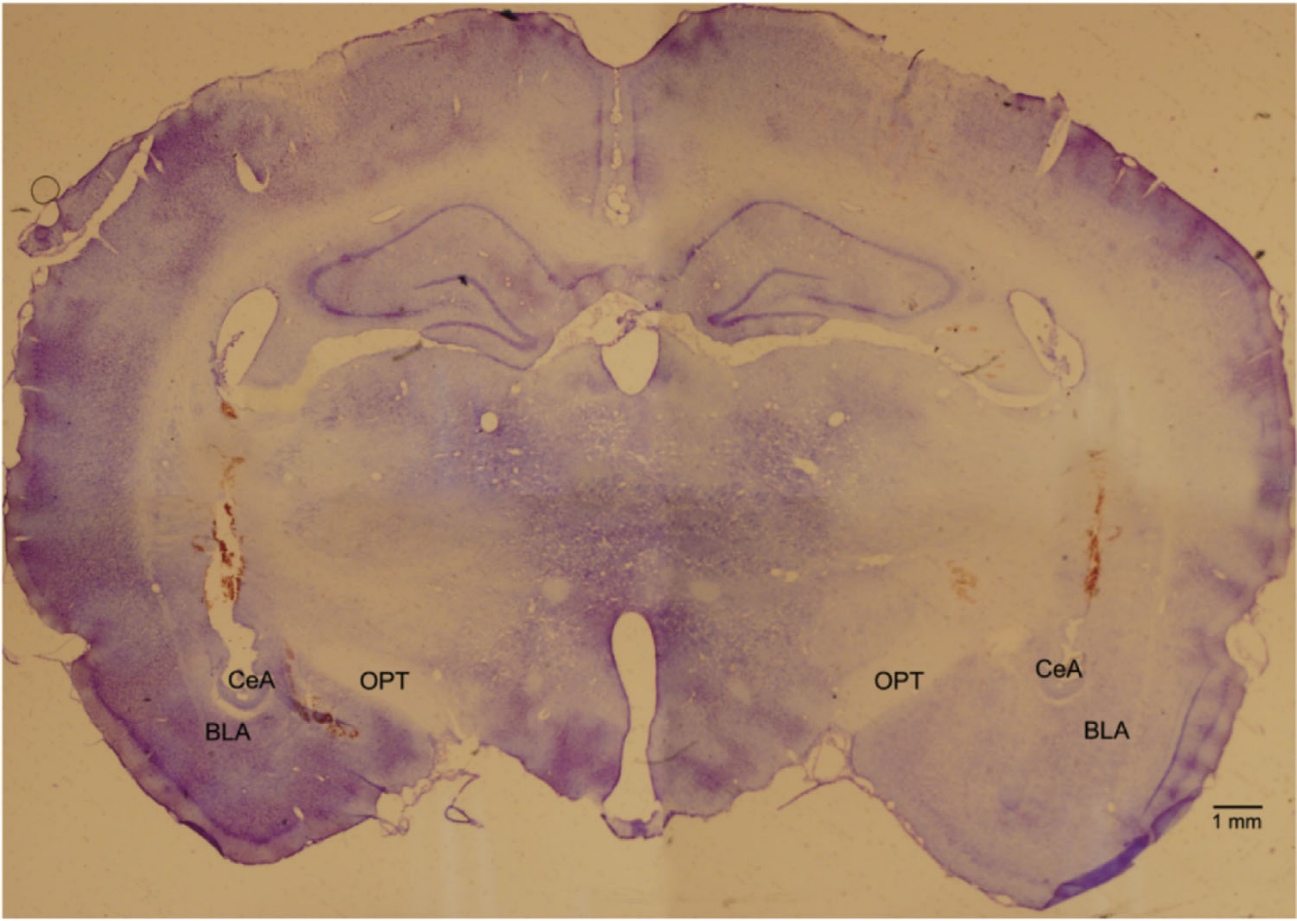
Photomicrographs of coronal rat brain sections showing the typical sites of bilateral electrolytic lesions of the CeA. Scale bar = 1 mm. Abbreviations: BLA, basolateral amygdala; CeA, central nucleus of amygdala; opt, optic tract

Typical bilateral electrolytic lesions in the CeA were observed, as shown in Figure 2. Bilateral CeA lesion sites were located at the dorsolateral side of the optic tract tip, the dorsal medial side of the basolateral amygdala, and the dorsal medial amygdala and medial amygdaloid nucleus. Of the 85 rats, all were subjected to CeA sham/unilateral/bilateral damage, of which 74 rats were damaged at accurate sites in 18 bilateral sham lesion rats and 19 unilateral sham lesion rats. Sixteen rats with bilateral sham damage and 16 rats with unilateral sham lesions were correctly injected into the solitary nucleus. In five sham lesion rats, injections were outside of the solitary nucleus. In 16 rats with bilateral lesions in the CeA and 16 rats with unilateral lesions in the CeA, the injections were accurately delivered into the solitary nucleus. In other experimental rats, one or two of the injections were outside of the solitary nucleus, with bilateral or unilateral lesions outside the CeA area or no apparent lesions in other parts of the brain. These data were not included in the statistical analysis.

### 3.2 Intake of 0.3 mol/L NaCl in rats with bilateral aldosterone injection in the solitary nucleus after electrolytic lesions in the CeA

**FIGURE 3:**
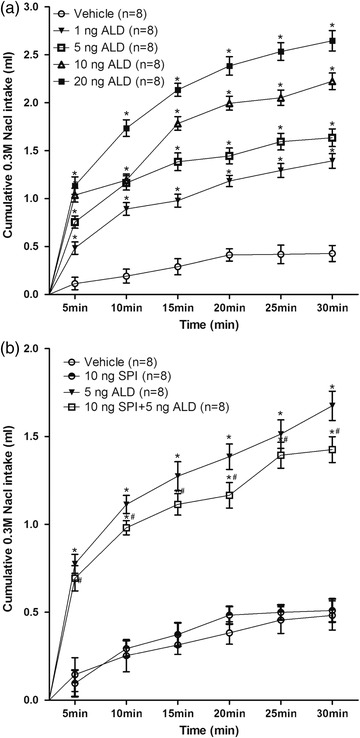
Cumulative intake of 0.3 mol/L NaCl by rats that received ALD injections at different concentrations into the nucleus tractus solitary (NTS) (a). Intake of 0.3 mol/L NaCl induced by sodium depletion followed by ALD or SPI treatment (b). *n* = number of animals. *p < .05, when each treatment group is compared with the vehicle group. Error bars show means ± SEM. ^#^ *p* < .05, when the treatment group is compared with the “10 ng SPI” group

The results showed an increase in sodium intake with increasing ALD concentration compared with vehicle, and the time course curves were significantly different between treatments [*F*(4, 35) = 45.76, *p <* .05)]. There was a peak in the first 5 min, which could be seen as an obvious rapid increase, as shown in Figure 3a. Compared with SPI group, the SPI + ALD group still stimulated sodium intake, which represented that SPI could not effectively block this rapid increase in sodium intake (Figure 3b), and this result indicated that this rapid regulation of ALD toward sodium intake may be a nongenomic effect.

**FIGURE 4:**
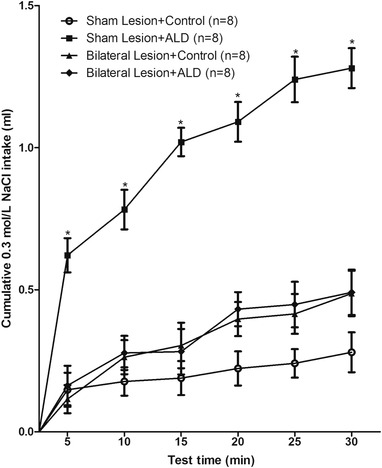
Cumulative 0.3 mol/L NaCl intake by rats submitted bilateral sham or electrolytic lesions of the central nucleus of the amygdala (CeA) that received bilateral injections of aldosterone (5 ng/0.1 μl) or control into the nucleus tractus solitary (NTS). *n* = number of animals. Error bars show means ± SEM. *p < .05, when each treatment group is compared with the vehicle group

A 5 ng/0.1 μl injection of aldosterone in the bilateral solitary nucleus significantly increased the intake of 0.3 mol/L NaCl in sham lesion rats, while electrolytic lesions in the CeA abolished the increased intake of 0.3 mol/L NaCl induced by aldosterone injection in the bilateral solitary nucleus (Figure 4).

**FIGURE 5:**
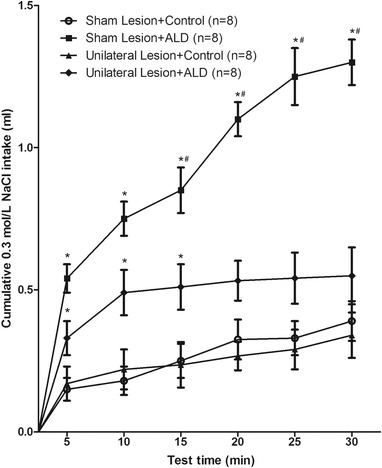
Cumulative 0.3 mol/L NaCl intake by rats submitted unilateral sham or electrolytic lesions of the central nucleus of the amygdala (CeA) that received bilateral injections of aldosterone (5 ng/0.1 μl) or control into the nucleus tractus solitary (NTS). *n* = number of animals. *p < .05, when each treatment group is compared with the vehicle group. Error bars show means ± SEM. ^#^ *p* < .05, when the treatment group is compared with the “unilateral lesion + ALD” group

Electrolytic lesions on the left side of the CeA reduced the higher intake of 0.3 mol/L NaCl induced by bilateral injection of 5 ng/0.1 μl aldosterone in the solitary nucleus. Compared with the control group, there was a significant difference in sodium intake in the first 15 min, with a difference that was not significant over the subsequent 15 min (*p *> .05). Compared with the unilateral lesion + ALD group, the sham lesion + ALD group showed a significant difference in sodium intake in the second 15 min (*p <* .05), as shown in Figure 5.

### 3.3 Change in intake of 0.3 mol/L NaCl in rats with electrolytic sham lesions in the CeA in which aldosterone was injected outside of the solitary nucleus

In rats with electrolytic sham lesions in the CeA, injections of 5 ng/0.1 μl aldosterone outside of the solitary nucleus (misplaced injection) failed to induce accelerated intake of 0.3 mol/L NaCl (*p *> .05).

## 4 DISCUSSION

The results demonstrate that electrolytic lesions of the bilateral CeA abolished the increased intake of sodium induced by aldosterone injections in both sides of the solitary nucleus, suggesting that there may be a facilitation mechanism in the CeA that is indispensable for aldosterone‐induced rapid sodium intake in rats.

The amygdaloid nucleus belongs to the limbic system of the forebrain, located on the dorsal medial side of the temporal lobe of the brain, the tip of the lateral ventricle, and the deep part of the parahippocampal sulcus. The amygdaloid nucleus is composed of a number of nuclei of varying size, and each subregion has unique characteristics of association (Janak & Tye, 2015; van den Burg & Stoop, 2018). As the critical component of the amygdaloid nucleus, the CeA receives ascending fiber projections of many nuclei in the brainstem, while it emits fiber projections to the dorsal nucleus of the vagus nerve, solitary nucleus, periaqueductal gray matter, and reticular nuclei of the pons. Recent studies have demonstrated that a close fiber link exists between the CeA and a number of regions of the brain associated with sodium intake regulation, such as signals from the HSD2 neuron (Geerling et al., 2006; Resch et al., 2017) in the solitary nucleus, relayed by the parabrachial nucleus, projected upward to the lateral CeA, and then projected directly by the medial CeA to the HSD2 neuron, which may be an important neural loop where the CeA participates in the regulation of sodium intake (Gasparini et al., 2017). In addition, the CeA has a fibrous connection with the nucleus of the reward system and emits fiber projections to neuroendocrine nuclei, such as the chamber nucleus. As the CeA has a two‐way fiber link with the brain stem group that transmits taste and visceral information and is closely related to the prebrain reward nerve loop and the peripheral organ, we hypothesized that the CeA plays a vital role in the regulation of sodium stabilization. Lateral ventricle injection of Ang II significantly enhances c‐Fos immunoreactivity in the CeA of rats (de Lucca Junior & Franci, 2004). Injection of glutamate into the CeA causes an increase in the frequency of discharge of Ang II‐sensitive neurons in the pre‐Kamakura region. Ventricle injections of renin or subcutaneous injection of psilocybe combined with 24‐h sodium‐free diet‐induced sodium intake resulted in an increase in sodium intake that was blocked by bilateral electrolytic lesions in the CeA. However, the CeA lesion did not affect the intake of water induced by subcutaneous injection of Ang II or dehydration of cells in rats, suggesting that CeA lesions specifically reduced sodium intake (Norgren et al., 2006).

Based on the data in this study, it can be concluded that unilateral lesions in the CeA eliminated the sodium intake increase induced by bilateral injection of aldosterone in the solitary nucleus, but the effects were different in unilateral and bilateral lesions of the CeA: bilateral lesions completely abolished the increase in sodium intake induced by aldosterone injection, while unilateral lesions in the CeA only partially reduced the increase in sodium intake. In rats with unilateral lesions in the CeA, aldosterone injection in the solitary nucleus still retained some ability of the intake of 0.3 mol/L NaCl in the first half of the experiment.

The mechanism by which CeA regulates sodium intake has been intensively studied in previous studies. In rats with electrolytic lesions on both sides, spontaneous sodium intake decreased significantly, and sodium intake induced by subcutaneous injection of oxidative corticosterone, α_2_‐adrenergic receptor antagonist, or Ang II was also reduced by creating a CeA lesion (Smith et al., 2016). Previous studies suggested that sodium appetite induced by various facilitation stimuli, such as Ang II and corticosteroids, depends on CeA facilitation mechanisms. The data in our study also demonstrate that the facilitation mechanism of CeA was indispensable for sodium intake induced by aldosterone in the solitary nucleus. However, compared with previous reports, we found that bilateral lesions of the CeA had no significant effect on sodium intake during the experiment. The exact reason for this difference remains unclear, and it is assumed that in this study, we observed sodium intake over a short period of time (30 min) rather than over the long term. Furthermore, the majority of previous studies used 0.5 mol/L NaCl as a sodium source, which is more aversive to rats than the 0.3 mol/L NaCl used in this study.

In summary, the results of this study indicated that CeA is indispensable for aldosterone's nongenomic effects of sodium intake in the solitary nucleus of rats. Complete CeA is required for aldosterone's nongenomic effects of rapid sodium intake. There may be a descending regulatory mechanism in the CeA in which aldosterone regulates sodium intake in the solitary nucleus.

## CONFLICT OF INTEREST

The authors declare no conflict of interest.

## AUTHOR CONTRIBUTION

Hu Qiao, Rui Wang, Nan Wang: Conceptualization, Funding acquisition, Investigation, Methodology, Project administration, Supervision. Wenhui Liang, Tingting Lin: Investigation, Methodology. Hu Qiao, Rui Wang: Roles/Writing original draft, Writing review & editing. All authors revised, edited and approved the final version of the manuscript.

### PEER REVIEW

The peer review history for this article is available at https://publons.com/publon/10.1002/brb3.2615

## Data Availability

Data that support the findings of this study and custom code used to analyze data are available from the corresponding author upon reasonable request.
